# 637. Burden to Healing: Equity in Trauma Care

**DOI:** 10.1093/jbcr/irag033.463

**Published:** 2026-04-06

**Authors:** Diana B Griffin, Robert Galiano

**Affiliations:** Northwestern University, Chicago, IL; Northwestern University, Chicago, IL

## Abstract

**Introduction:**

Trauma and injury burden remains a major public health challenge with substantial variation in disability and outcomes across sociodemographic strata and geographic regions. Disparities in access to surgical care result in delayed treatment, higher complication rates, and worse outcomes, which are reflected in increased disability-adjusted life-years (DALYs). Consensus frameworks propose that population-level DALY curves can be used to estimate the impact of expanding surgical care.

This study aims to quantify disparities in trauma-related DALY burden across sociodemographic and regional groups in the United States, and further aims to address burn specific impact to inform equitable surgical care and resource allocation.

**Methods:**

This is a systematic analysis referencing the Global Burden of Disease 2021 study (1990-2021). The study examined DALYs and sociodemographic index (SDI) across ages and U.S. regions for four injury causes: transport, mechanical forces, interpersonal violence, and fire/heat. Specific sub-analysis investigated the outcomes for the fire/heat injuries in comparison to the other forms of trauma. Data analyses utilized two-way anova, one-way anova, tukey test, and t-tests.

**Results:**

As communities move from lower to higher SDI, trauma DALY burden generally declines with a gradient dependent on age and region. The trends described for the trauma group at large generally hold for burn specific trauma; however, burns proved to be less SDI (r = -0.09, *p*=.002) and region (*p*=.12) sensitive and have less DALY contribution. SDI matters in every age group for trauma, especially at the lowest and highest SDI quartiles (largest age effects in Low/High; partial n^2 = 0.10 and 0.09). Regionally, the West shows a clear step down with each higher SDI level (Low vs High = -6337 DALYs; Tukey *p*<.001). Additional longitudinal analyses (Fig. 2) demonstrate strong region and year effects with modest interaction (partial n^2 up to 0.58) and region differences detectable in every year. Since 1990, burns show the largest and most consistent long-run improvements.

**Conclusions:**

DALY falls with increasing SDI, yet persistent disparities by age, region, and cause – largest in low-SDI strata – translate into losses of thousands of healthy life-years. Targeted prevention and expansion of surgical capacity in low-SDI communities and high-gap regions should be prioritized across all forms of trauma.

**Applicability of Research to Practice:**

These results represent gaps of thousands of DALYs, which are policy-relevant. Stratifying outcomes by sociodemographic and regional indices allows clinicians and policymakers to identify where gaps are greatest, enabling more precise outreach, referral pathways, and investment. Integrating DALY-based monitoring into practice can inform quality improvement initiatives and strengthen advocacy for equitable distribution of burn care resources across the U.S.

**Funding for the study:**

N/A.

